# Stress fractures

**DOI:** 10.20945/2359-3997000000562

**Published:** 2022-11-10

**Authors:** Tatiana Munhoz da Rocha lemos Costa, Victoria Zeghbi Cochenski Borba, Renata Gonçalves Pinheiro Correa, Carolina Aguiar Moreira

**Affiliations:** 1 Universidade Federal do Paraná Curitiba PR Brasil Universidade Federal do Paraná (UFPR), Curitiba, PR, Brasil; Serviço de Endocrinologia do Hospital de Clínicas da UFPR (SEMPR), Curitiba, PR, Brasil; UFPR Hospital de Clínicas Serviço de Endocrinologia Curitiba PR Brasil; 2 Universidade Federal do Paraná Departamento de Clínica Médica Brasil Departamento de Clínica Médica, Universidade Federal do Paraná (UFPR); Unidade de Ossos do Serviço de Endocrinologia do Hospital de Clínicas da UFPR (SEMPR), Curitiba, PR, Brasil; Ex-bolsista da Unidade de Ossos da Universidade de Arkansas para Ciências Médicas (UAMS), Arkansas, USA; UFPR Hospital de Clínicas Unidade de Ossos do Serviço de Endocrinologia Arkansas USA; 3 Universidade Federal do Paraná Curitiba PR Brasil Universidade Federal do Paraná (UFPR), Curitiba, PR, Brasil; 4 Universidade Federal do Paraná Centro Acadêmico de Pesquisa do Instituto Pró-Renal Departamento de Clínica Médica Curitiba PR Brasil Departamento de Clínica Médica, Universidade Federal do Paraná (UFPR); Centro Acadêmico de Pesquisa do Instituto Pró-Renal (PRO), Curitiba, PR, Brasil

**Keywords:** Stress fractures, athlete triad, RED syndrome, overtraining, magnetic resonance

## Abstract

Stress fractures (SF) represent 10%-20% of all injuries in sport medicine. An SF occurs when abnormal and repetitive loading is applied on normal bone: The body cannot adapt quickly enough, leading to microdamage and fracture. The etiology is multifactorial with numerous risk factors involved. Diagnosis of SF can be achieved by identifying intrinsic and extrinsic factors, obtaining a good history, performing a physical exam, and ordering laboratory and imaging studies (magnetic resonance imaging is the current gold standard). Relative energy deficiency in sport (RED-S) is a known risk factor. In addition, for women, it is very important know the menstrual status to identify long periods of amenorrhea in the past and the present. Early detection is important to improve the chance of symptom resolution with conservative treatment. Common presentation involves complaints of localized pain, with or without swelling, and tenderness on palpation of bony structures that begins earlier in training and progressively worsens with activity over a 2- to 3-week period. Appropriate classification of SF based on type, location, grading, and low or high risk is critical in guiding treatment strategies and influencing the time to return to sport. Stress injuries at low-risk sites are typically managed conservatively. Studies have suggested that calcium and vitamin D supplementation might be helpful. Moreover, other treatment regimens are not well established. Understanding better the pathophysiology of SFs and the potential utility of current and future bone-active therapeutics may well yield approaches that could treat SFs more effectively.

## INTRODUCTION

Stress fractures (SFs) were initially described in the mid-19th century in military personnel due to excessive training in the recruitment period. SFs can also occur in athletes or during sports activities. Normal bone is constantly remodeled and adapted to the loads placed on it. SFs occur when abnormal and repetitive loading is applied on normal bone: The body cannot adapt quickly enough, leading to microdamage and fracture. Usually, symptoms appear 3 weeks after a change in physical activity. Symptoms increase progressively, culminating in failure of loading or the need to stop the physical activity. SFs should be differentiated from insufficiency fractures that result from normal load on a pathological bone (1,2). This narrative review will explore the main aspects of SFs, including risk factors, pathophysiology, evaluation, and treatment.

### Epidemiology

The epidemiology of SFs varies with the type of activity; it is higher in military training and increases with longer periods of training. The SF rate during basic army training is between 0.9% and 5.2% for males and 3.4% and 21.0% for females over 8 weeks. In mariners, the prevalence in 12 weeks of basic training is 0.8% to 4.0% in males and 3.0% to 5.7% in females. In the Israel Defense Forces, in 4 years of study of 62,371 soldiers (10.1% women), 3,672 (5.9%) were diagnosed with clinical SFs, on average 21 days after the beginning of training (1).

Women have a higher rate of fractures. The incidence of SF in the US Army, is 19.3 male and 79.9 female cases per 1,000 recruits within the 10 weeks of basic training (3,4).

SFs represent 10%-20% of all injuries in sport medicine and 10% of all orthopedic injuries (6,7). The SF rate is 1.54 per 100,000 athletes-exposure, and around 0.8% of high school athletes sustain an SF (2,7). In addition, females in sports have more injuries than males do, and one in seven athletes has a history of SF. The rate of SF in sex-comparable sports is 2.22/100,000 in girls and 1.27/100 000 in boys (7). The prevalence is also different according to the type of sport, being more frequent in endurance runners, track-and-field athletes, and dancers (8). SFs represent 15%-20% of all musculoskeletal-related injuries in runners (9), and 22% in female track-and-field athletes (5,6).

Lower extremities are the most affected site (8%-95%); the upper extremity accounts for less than 10% of SFs (6). The order of prevalence is tibia (49%), tarsal bones (25%), metatarsals (9%), femur, and fibula (6,10). The ulna is the bone most affected in the upper extremity. Location varies by sport (Table 1 summarizes the locations of SFs by sport).

**Table 1:** Stress fracture by type of sport (11)

Sport	Location
Track and field athletes	navicular, tibia, and metatarsals
Distance runners	tibia and fibula
Dancers	metatarsals
Military recruits	calcaneus and metatarsals

### Pathophysiology

SFs reflect an imbalance between bone strength and the mechanical load placed upon the bone. When abnormal stress is applied to a normal bone, a fatigue fracture can occur, but when normal stress is applied to an abnormal bone, an insufficiency fracture occurs. The population, sites, and pathophysiology differ between them (6,11). Bone follows Wolff's law: Upon stress, it deforms through the bone's elastic range and returns to its initial conformation if the stress stops. However, stress that persists beyond the elastic range creates microfractures and a persistent plastic deformity. When microfractures cannot be repaired by remodeling, they coalesce into a discontinuity within the cortical bone and a fracture occurs. SFs are the result of a disbalance between the remodeling and microdamage, leading to inadequate repair and cumulative damage, with predominance of osteoclastic activity over osteoblastic activity and new bone formation (11–13).

### Etiology

**Figure 1:**
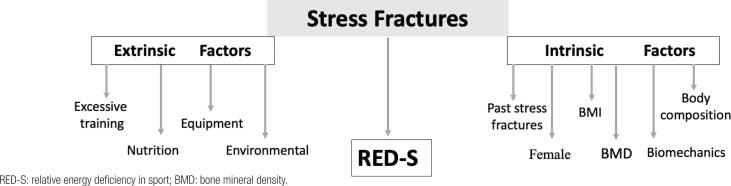
Main risk factors for stress fractures.

The etiology of SFs is multifactorial with numerous risk factors involved. Identifying the risk factors helps to characterize individual susceptibility to developing SFs, and it could indicate strategies for prevention. Figure 1 summarizes the main risk factors.

#### Extrinsic factors

1. Nutritional deficiencies (e.g., low intake of calcium and vitamin D deficiency) were previously related to SF (2,6,11).
2. Eating disorders or other psychopathologies related to eating, body image, and exercise have been identified amongst female athletes with SF. However, eating disorders have not been found to be an independent predictor of increased risk of SF (6), but athletes may have unhealthy eating habits, poor body image, or compulsive exercise training that predispose them to SFs. Indeed, eating disorder is one of the components of the athlete's triad.
3. Type and frequency of activities are important risk factors for SF; for example, repeated submaximal stresses (running, jumping, or marching); high-impact loading, new or excessive exercise, change in the type or intensity of the activity, and limited rest following excessive physical activity. Runners with SF exercised 3 more hours per week compared to runners who did not have SF, and dancers who practiced 6 or more hours per day had more SFs (2,6,13).
4. Quality of footwear and equipment as well as environmental factors such as the running surface also affect the rate of SF (2,13).

#### Intrinsic factors

The intrinsic factors are individual factors that predispose a person to SF.

1. Having a past SF increases the chances of a new SF in female runners by 5-6 times. SFs are associated with structural changes (bone geometry, running mechanics, and lower bone strength) that predispose an individual to a fracture (6,11,14).
2. Female individuals are more susceptible to SF; a meta-analysis of distance runners showed 2.3 times greater risk of SF in females, explained in part by decreased lean mass and less robust bone morphology (6,13,14).
3. Body mass index (BMI) has an indirect relationship with SFs. BMI < 19 kg/m^2^ seems to be the threshold for SF, but BMI > 30 kg/m^2^ was also related to SF (15,16). Women who lose more weight during training have a higher rate of SF, related to negative energy balance and lower baseline physical fitness with muscle fatigue, less dissipation of energy by muscles, and more energy absorption by bone (6,17). Females with SFs have two profiles: one with low BMI at risk of the athlete's triad and another in high-BMI athletes with lower fitness (6).
4. Body composition: Lower lean body mass and higher fat mass are associated with an increased risk of SF in athletes and nonathletes due to increased load and bone stress, with early fatigue (6,18,19). This observation could explain why female athletes are at higher risk of SF, because women generally have higher fat mass and lower lean mass than men have. However, a recent study did not confirm the influence of body composition on SF (20). When athletes are oligo/amenorrheic, it seems the body composition loses importance (21).
5. Bone mineral density (BMD): The importance of BMD to SF is controversial, with studies showing correlation of low BMD with higher rates of SF (6,9,21) and others not (6,22), although oligo/amenorrheic athletes with SF had lower whole body and spine BMD (21). Postmenopausal women fracture at a higher T-score than premenopausal women with SF do (23).
6. Biomechanics: The combined effects of morphological variation and malalignment of bone, muscle, and joint dynamics influence the development of SF, especially in foot and ankle. Their effect depends on the type of physical activity (2,6). For example, cavus foot could lead to SF of the femur and metatarsal bones, whereas flat foot increase pronation and SF of the tibia, the fibula, and the tarsal bones. Varus alignment in the lower limb increases SF risk of the femur and the tarsal bones, and cavovarus feet have a rigid foot shape that does not attenuate the impact and predisposes a person to SF (10,24).

Athlete's triad or relative energy deficiency in sport (RED-S) is a known risk factor for SF. RED-S is characterized by the presence of eating disorders or low energy availability, amenorrhea or menstrual dysfunction, and changes in BMD or osteoporosis. Not all elements are necessary for the diagnosis; the combinations vary and depend on the type of sport. Energy deficiency is the key factor affecting several physiological functions with consequences for an athlete's performance and health (25,26). Low energy availability causes estrogen deficiency and hormonal changes in cortisol and leptin levels that affect bone health, leading to low BMD. The presence of the three components is seen in 1%-14% of female athletes, but up to 78% of female athletes have at least one aspect of the triad at a given time (26). The risk of SF increases with the increment of number of components present; it goes from 15% to 20% for athletes with a single risk to 30%-50% for those exhibiting multiple risk factors (27,28). Male athletes, including cyclists, rowers, runners, jockeys, and athletes in weight-class combat sports are also at risk for RED-S and SF. For males, besides the type of sport, risk factors include cyclical changes in body mass and composition (i.e., “making weight”), prolonged inadequate energy intake to meet the high energy expenditure of endurance sport, punctuated changes in training volume/intensity, and participation in strenuous endurance events without accompanied changes in nutrition (25).

### Diagnosis

Diagnosis of SF can be achieved by identifying intrinsic and extrinsic factors, obtaining a good history, performing a thorough physical exam, and ordering laboratory and imaging studies. Early detection is important to improve the chance of symptom resolution with conservative treatment (29).

#### History

Common presentation involves complaints of localized pain, with or without swelling, and tenderness on palpation of bony structures that begins earlier in training and progressively worsens with activity over 2-3 weeks. The pain is often exacerbated by repetitive loading; over time, the pain may progress until it is also present with ambulation and in rest (16,29). A detailed history of the onset of the pain and the identification of related extrinsic factors associated with physical exam are the first step in the diagnosis of SFs. It is important to characterize whether the physical activity load increased suddenly or whether the rest between training sessions was inadequate, which helps a clinician to think about a bone injury related to the exercise (13–16). Further, questions should address the underlying causes of SFs, such as history of SF, dietary history including calcium and vitamin D intake, medication list, treatment history, other diseases such as eating disorders, depression, endocrinopathies, autoimmune diseases, malabsorption, and bariatric surgery (29). For women, it is very important know about the menstrual status by identifying past or present long periods of amenorrhea (30–31). It is important to consider differential diagnoses such as neoplasm, tendinitis, infection, periostitis, exertional compartment syndrome, osteoid, osteoma and intermittent claudication (10).

#### Physical exam

On exam, the clinician will appreciate focal tenderness on the area of a suspected stress injury. Soft tissue swelling may occur (11). Soft tissue sensitivity tends to suggest muscle injury, whereas bony tenderness is more likely to suggest SF. For areas in which a suspected fracture would be difficult to palpate, such as the femoral neck, it is important to evaluate for pain with range of motion of the joint. The pelvis and sacrum require the clinician to have a higher index of suspicion from the history alone (11–29).

In addition, tests can be used to evaluate SFs. The hop test can be used to distinguish tibial SFs from medial tibial stress syndrome. Patients with stress injuries can tolerate repeated jumping, whereas patients with SFs cannot hop without pain (11). The 3-point “fulcrum test” can be used to aid in the diagnosis of femoral and tibial SFs. The examiner's arm is used as a fulcrum under the thigh while pressure is applied to the knee. A positive test is pain or apprehension at the point of the fulcrum. Sacral SFs can be assessed with the FABER (Flexion, ABduction, and External Rotation) and/or Flamingo (stand on one leg and hop) tests (32).

#### Imaging

Despite the suggestive history and the local symptoms, SFs are generally confirmed through image exams such as radiographs (X-ray), magnetic resonance imaging (MRI), computed tomography (CT), or bone scintigraphy (bone scans) (29).

#### Radiographs

Because of their low cost and high availability, plain radiographs are frequently obtained at first. However, radiographs are usually negative (70%) in early SFs and tend to become positive at approximately 3 weeks. Cortical radiolucency is the earliest radiographic finding, with poor cortex definition signifying the fracture site (6,8). Periosteal new bone formation and linear sclerosis may be seen before a fracture line is visible; endosteal thickening is seen weeks to months later, indicating new bone formation. Either of these findings supports the diagnosis of SFs (6,8,29). If pain persists after conservative treatment despite normal X-rays, an MRI, CT, or bone scan may be necessary.

#### Magnetic resonance imaging

**Figure 2:**
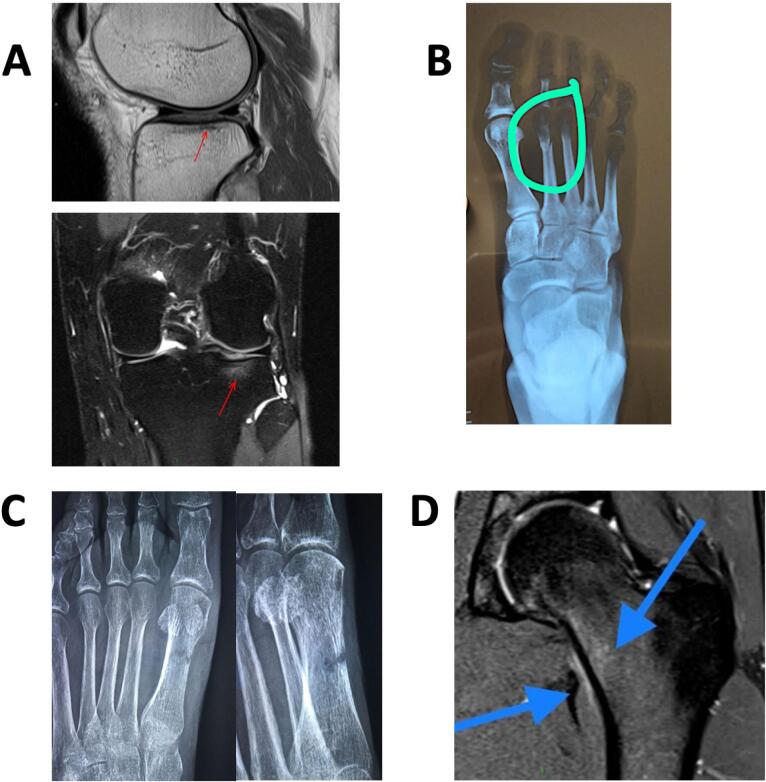
Images of stress fractures. **A:** 37-year-old male with knee pain after walking 5 km; **B:** 23-year-old female with oligomenorrhea, BMI of 16 kg/m^2^, pain after 10 km running; **C:** 28-year-old male after increased training for a running competition; **D:** Grade 1 SF based on MRI findings and using the Fredericson classification system (see Table 2) (33).

MRI is the most sensitive (approximately 88%) and specific diagnostic image for SF, and it is the current gold standard for diagnosing SFs (3,4,7–9,11) (Figure 2). In fact, MRI can identify both soft tissue and bone edema, the early sign of SF that can be seen at 1 or 2 days after the onset of the bone pain (34).

**Table 2:** Fredericson classification according MRI findings of stress fracture

Lesion Stage	MRI Finding
Grade 1	Periosteal edema only
Grade 2	Bone marrow edema (only on T2)
Grade 3	Bone marrow edema on T1 and T2
Grade 4^a^	Multiple discrete areas of intracortical signal changes
Grade 4b	Linear areas of intracortical signal change correlating with a frank stress fracture

#### Computed tomography

CT presents high specificity for SF but relatively low sensitivity, around 42% (34). However, in certain situations where MRI is not available or contraindicated, CT may be useful to differentiate between complete and incomplete fractures and to identify SFs in specific bones such as the sacrum, pelvis, and spine (6,29,30).

#### Bone scintigraphy

Bone scintigraphy is moderately sensitive at 74%. It can show signs of fracture 3 to 5 days after the onset of the local symptoms, especially in states of increased bone remodeling. Bone scintigraphy is particularly useful when patients are suspected of having multiple SFs simultaneously. However, increased uptake can also be due to other pathologies (avascular necrosis, osteomyelitis, and neoplasm, among others), which makes specificity low (33).

### Bone mineral density

Studies had evaluated BMD in subjects who presented SFs to examine whether, in addition to the effect of repetitive stress on the skeleton, a reduction in bone mass occurs. Lauder and cols. were the first to evaluate BMD in women army soldiers and found a strong negative relationship between the probability of SFs and femoral neck BMD (35).

Studies have evaluated BMD in adolescent athletes with SFs and demonstrated lower spine and whole-body BMD in both males and females (36,37). Interestingly, structural bone parameters of the tibia, measured by peripheral quantitative computed tomography (QCT), demonstrated more deterioration in bone structure in healthy military personnel with SF when compared to those without fractures (38). These findings suggest that bone quality may be compromised in individuals who develop SFs and therefore it is very important to avoid recurrent SFs. In clinical practice, a bone density test is indicated for subjects who present recurrent SFs (13).

#### Laboratorial exams

A basic chemistry panel along with measurement of 25OH vitamin D, thyroid function tests, and 24 hour urinary calcium excretion has been proposed to evaluate subjects with recurrent SFs (13). Both evaluation of vitamin D status through measurement of 25-hydroxivitamin D [25(OH)D] and treatment of hypovitaminosis D is recommended because a study has shown that higher doses of 25(OH)D reduce the chance of developing tibial or fibula SFs in white female navy recruits (39). In a prospective study in male military recruits, 25(OH)D levels did not differentiate fracture cases from others. However, there was a significant association of SFs with higher parathyroid hormone (PTH) levels. In fact, their findings showed that serum levels of PTH were 60% higher in those with fractures (39).

The relationship between biochemical markers of bone turnover and SFs was evaluated in a few studies. Välimäki and cols. (40) found that bone turnover marker levels were similar in men with and without SFs. Similarly, another study (41) showed no difference in serum cross-linked collagen telopeptide concentration between female recruits with SFs and matched controls at any stage of training.

Bioavailable serum insulin-like growth factor 1 (IGF-1) levels decreased during basic training among women with SFs, whereas women without fractures had increased bioavailable IGF-1. These findings suggest that IGF-1 concentrations per se and/or their response to physical training may help to account for SF susceptibility (41).

#### Classifying stress fractures

Appropriate classification of SFs based on type, location, grading, and low or high risk is critical in guiding treatment strategies and influencing the time to return to sport. Several grading schemas have been developed for specific injury sites based on image findings. Using schemas has allowed better determination of expected recovery time, which is a clinically useful parameter (42). One of the grading schemes is based on bone scintigraphy and MRI (Table 3) (29).

**Table 3:** Classification of stress fracture as low and high grade based on bone scintigraphy and MRI

Grade	Bone scintigraphy findings	MRI findings
Low	Irregular uptake and/or a poorly defined area of increased activity, compared with the contralateral side	Bone marrow edema in STIR images, possibly in T2-weighted images
High	Sharply marginated area of increased activity, compared to the other side, usually focal or fusiform in shape	Bone marrow edema on T1- and T2-weighted image with or without a fracture line

The anatomic location of an SF is used to classify the injury as low or high risk. Low-risk SFs have decreased chance of lower recurrence rates, low risk of complication, and poor healing. Mutually, high-risk sites have a greater likelihood for fracture propagation, nonunion, or delayed union (43). Risk level classification depends on the local blood supply and the tension or compression inherently applied to the specific location of the SF (Table 4) (43).

**Table 4:** Risk classification by anatomic site of stress fractures

Risk Classification	Site
Low	Calcaneus, fibula, femoral shaft, first through fourth metatarsals, posterior/medial tibia, first metatarsal sesamoids, pelvis, ribs, ulnar shaft
Medium	Femoral shaft, pelvis, posterior/medial tibia, fifth metatarsal
High	Anterior tibia, femoral head, femoral neck, fifth metatarsal, first metatarsal sesamoids, navicular, medial malleolus, patella

### Treatment

Treating SFs requires a multidisciplinary approach to address all potential causes of the injury (29). Treatment of stress injuries varies depending on whether it is a stress reaction or SF, by the site of injury, and by its suitability for rehabilitation (11). Stress injuries at low-risk sites are typically managed conservatively with a two-phase protocol. The first step should be the cessation of sport activity for 6-8 weeks, along with pain relief medications (12). However, most athletes could be encouraged to do alternative lower load exercise in the meantime, such as hydrotherapy or swimming, anti-gravity treadmill cycling, and elliptical workouts to maintain strength and fitness and to minimize immobilization-induced muscle wasting, helping to ease the return to training (42). If a patient cannot ambulate without pain, temporary immobilization is indicated. Phase 2 begins after a period of pain-free rest of 10 to 14 days and involves a gradual return to activity over the subsequent weeks, including continued physical therapy. Formation of bone callus as well as obliteration of the fracture line seen on radiographs, MRI scans, or CT scans may help to establish recovery (11,13).

Targeted actions with respect to several extrinsic factors, such as footwear and training surfaces, might be helpful. Athletes with overly pronated or supinated feet may benefit from orthotics. Running shoes should be changed every 300 to 350 miles of use depending on the type of shoe, surface, and athlete (44).

#### Nutritional and medical therapy

An increase in calcium intake and vitamin D sufficiency is recommended and was reinforced after Lappe and cols.'s study demonstrated that higher intake of calcium (2 g/day) was associated with lower incidence of SFs in soldiers (45). This finding indicates that the mechanism of bone repair in response to the repetitive stress from exercise needs a local positive calcium balance and therefore a higher calcium intake would help in the prevention of SFs. If a patient's intake of calcium were inadequate to account for this generous intake, then supplementation would be necessary (45).

A systematic review and meta-analysis by Dao and colleagues (46) examined the association between serum 25(OH)D levels and SFs specifically in the military. The analysis included 2634 military personnel, with 761 SF cases and 1,873 controls. The authors found that the overall mean serum 25(OH)D level was significantly lower for SF cases than it was for controls. In an interventional trial involving female navy recruits, the participants were randomized to supplementation with 2,000 mg calcium and 800 international units of vitamin D versus placebo for 8 weeks of basic training. Those who had supplementation had a 20% lower incidence of SF (44).

It is not known whether an optimal 25(OH)D level for athletes differs from that of the overall population. Shuler and cols. (47) recommended athletes supplement with 25(OH)D below 30 ng/mL. The Female Athlete Triad Coalition recommended maintaining levels between 32 ng/mL and 50 ng/mL (48).

Few studies have demonstrated that bisphosphonates may be useful for improving pain and helping to shorten the return to activities (49). In five cases, intravenous pamidronate was reported to be effective in reducing the time needed before returning to training after an SF (13). In a retrospective study, the safety and efficacy of intravenous ibandronate and high-dose vitamin D were evaluated for bone marrow edema syndrome and SF in 25 high-performance athletes. After ibandronate administration, reduction of pain at rest and under strain along with improved mobility were reported in 64% of subjects within 2 weeks. For those who had an early diagnosis and rapid onset of treatment, the time needed before returning to activities was shortened (49). A study of military recruits showed that risedronate for 12 weeks was not effective in reducing SF incidence, delaying time to onset, or decreasing the severity of fractures (50). Bisphosphonates are not approved for this indication by the US Food and Drug Administration.

The use of anabolic agents such as teriparatide is of interest to treatment of SFs because this medication may accelerate fracture healing. In animal models, teriparatide has been shown to improve bone mineral content, callus volume, and rate of successful union at fracture sites (51). Several case reports have demonstrated enhanced healing with teriparatide treatment in patients with delayed fracture healing (52–54). Recently, a panel of experts published a consensus statement on fracture healing and considered the use of teriparatide for fracture healing as having an efficacy level of grade 7 on a scale of 1 to 9. This minimal consensus agreement is undoubtedly due to the paucity of clinical trials and the need for more evidence (13). No study has specifically been conducted to evaluate the effect of teriparatide on SFs, but a clinical trial is aiming to evaluate it (55).

Future treatments such as antisclerostin agent could accelerate fracture healing. Analogs of parathyroid hormone, such as teriparatide, might be relevant to the hypothesis that fracture healing is accelerated. New drugs used in the treatment of postmenopausal osteoporosis may show promise. The RANKL inhibitor denosumab is a candidate by virtue of its positive effect on cortical bone. Moreover, newer osteoanabolic drugs, such as abaloparatide, an analog of parathyroid hormone-related protein, and romosozumab, an antibody against sclerostin, may give hope (13).

#### Prevention

Preventing SFs is critical. Education of health professionals, coaches, and athletes is necessary to ensure early diagnosis and treatment. Both extrinsic and intrinsic risk factors associated with such injuries must be considered. Screening for the female athlete triad is useful for addressing and correcting low energy availability and disordered eating, which can lead to menstrual dysfunction. Screening at-risk female athletes early can help improve bone health over time.

SF patients should ensure good nutrition, including calcium, vitamin D, and adequate protein, and avoid negative energy balance. Increases in an exercise regimen should be conducted gradually and, in certain circumstances, under supervision (13,29).

In conclusion, SFs are common among athletes and military recruits, and understanding the identification, classification, diagnosis, treatment, and preventative measures is necessary to maximize positive outcomes and minimize morbidity. Early diagnosis is crucial and relies on a thorough history, physical exam, and evaluation using appropriate imaging modalities. Protocols for management of SF, including investigation, preventive strategies, and treatment, are lacking in the literature.

Moreover, treatment regimens are not well established. Understanding better the pathophysiology of SFs and the potential utility of current and future bone-active therapeutics may well yield approaches that could treat SFs more effectively.
